# Linoleic acid–good or bad for the brain?

**DOI:** 10.1038/s41538-019-0061-9

**Published:** 2020-01-02

**Authors:** Ameer Y. Taha

**Affiliations:** Department of Food Science and Technology, College of Agriculture and Environmental Sciences, University of California, Davis, CA 95616 USA

**Keywords:** Neurochemistry, Oils

## Abstract

Increased intake of omega-6 rich plant oils such as soybean and corn oil over the past few decades has inadvertently tripled the amount of n-6 linoleic acid (LA, 18:2n-6) in the diet. Although LA is nutritionally “essential”, very little is known about how it affects the brain when present in excess. This review provides an overview on the metabolism of LA by the brain and the effects of excess dietary LA intake on brain function. Pre-clinical evidence suggests that excess dietary LA increases the brain’s vulnerability to inflammation and likely acts via its oxidized metabolites. In humans, excess maternal LA intake has been linked to atypical neurodevelopment, but underlying mechanisms are unknown. It is concluded that excess dietary LA may adversely affect the brain. The potential neuroprotective role of reducing dietary LA merits clinical evaluation in future studies.

## Introduction

Linoleic acid (LA, 18:2n-6) is an essential n-6 polyunsaturated fatty acid (PUFA)^1^ required for normal growth and development at 1 to 2% of daily energy.^2^ LA has become ubiquitous in Western diets over the past few decades due to agricultural shifts towards high-LA soybean and corn oils during the late 1930s, resulting in a greater than 3-fold increase in intake.^3,4^ Historic levels of LA intake ranged between 1 to 2% of daily calories pre-1930s, but currently average more than 7% of daily calories.^4^ Based on economic disappearance data, the majority of LA in the US diet comes from soybean oil.^4^

Amongst other dietary PUFAs important for optimal health, LA is the only one that has markedly changed in the diet over the past few decades. Contrary to common misconception, the intake of LA’s elongation-desaturation product, arachidonic acid (AA, 20:4n-6), and the essential n-3 fatty acid, alpha-linolenic acid (ALA, 18:3n-3), and its elongation-desaturation products eicosapentaenoic acid (EPA) and docosahexaenoic acid (DHA), has remained relatively constant at <1% energy since the early 1900s.^4^ Excess LA in the food supply has shifted the n-6 to n-3 ratio from 4:1 to 20:1.^4,5^

Surprisingly, little is known about how this increase in dietary LA affects the brain. It is mainly viewed as an essential precursor to AA, which is important for neurodevelopment and other physiological processes.^6^ Despite being a major part of the diet, LA has been considered non-functional in the brain because of its low concentration (<2% of total fatty acids) compared to palmitic acid (16:0), stearic acid (18:0), oleic acid (18:1n-9), DHA, and AA which make up over 84% of brain fatty acids in rats and humans.^7,8^

LA is known to enter the brain at a rate of ~4 pmol/g/s, which is comparable to the rate of entry of AA, DHA, and other fatty acids into the brain.^9,10^ However, unlike AA and DHA which mostly incorporate into brain membrane phospholipids, the majority (~59%) of LA entering the brain is rapidly β-oxidized into aqueous products, likely composed of carbon dioxide, acetate, and other polar metabolites of β-oxidation.^9^ Some of the 2-carbon acetate molecules produced by β-oxidation are recycled into cholesterol via de novo lipid synthesis pathways within the brain.^9,11^

LA is also a precursor to oxidized products known as ‘oxidized linoleic acid metabolites’ (OXLAMs) that are produced by auto-oxidation or enzymatically via lipoxygenase (LOX), cyclooxygenase (COX), cytochrome P450 (CYP450), and soluble epoxide hydrolase (sEH).^12–15^ OXLAMs are lipid mediators known to regulate pain and inflammatory signaling in peripheral tissue,^16–19^ where they are abundant.^18,20^ In the brain, they are presumed to be formed by LOX, COX, CYP450, and sEH enzymes, but their role there is not fully understood.

This review highlights the current state of knowledge on the role and metabolism of LA and OXLAMs in the brain. Studies linking LA and OXLAMs to reported biochemical, neuropathological, or behavioral endpoints in chickens, rodents, and humans are discussed, and directions for future research are proposed.

## Effects of LA or OXLAMs on chickens

Studies in the late 1950s and 1970s showed that chicks fed a vitamin (V)-E deficient diet containing LA developed encephalomalacia,^21–25^ a neurodegenerative condition characterized by necrosis, edema, and microvasculature abnormalities that can lead to ataxia and death.^26^ Table 1 provides a detailed summary of all these studies, which are discussed herein.

**Table 1:** Effect of LA or OXLAMs on neurological outcomes in chickens.

Reference	Animal model	Age/sex (male, female)	Diet	Duration	Outcome
Dam et al.,^21^	Chicks	2-day/unknown	V-E-free diet V-E-free diet + 1.5% ethyl-LA V-E-free diet + 1.5% ethyl-ALA	28 days	11/12 chicks died from encephalomalacia in the Vitamin E free + 1.5% ethyl-LA group, versus 0/12 in the other 2 groups
Dam et al.,^24^	Chicks	~6-days/unknown	V-E free diet + 30% lard (source of AA) V-E-free diet + 1.5% ethyl-LA V-E-free diet + 1.5% ethyl-AA	35 days^a^	All chicks in lard group developed encephalomalacia by 29 days. All chicks in ethyl AA group developed encephalomalacia with 13 days. Only 70% in ethyl-LA group developed encephalomalacia by 35 days
Bartov and Bornstein,^23^	White rock hens	30 months/female	Maternal diet had 4% soybean oil or tallow with 5 or 25 mg α-tocopherol acetate. Chick diet contained 4 or 10% oxidized safflower oil^b^.	Up to 31 days of age	Increased incidence of ataxia and mortality in chicks born to mothers on a 4% soybean oil diet with low (5 mg) vitamin E content
Budowski et al.,^25^	Chicks	1-day/male	Exp 1 (20 chicks per group): V-E free diet + 4% oxidized safflower oil^b^ V-E free diet + 4% safflower oil methyl esters	3 weeks	35% ataxia; 25% mortality 75% ataxia; 45% mortality
			V-E free diet + 10% oxidized safflower oil^b^ V-E free diet + 10% safflower oil methyl esters	2 weeks	60% ataxia; 45% mortality 40% ataxia; 5% mortality
			Exp 2 (20 chicks per group): V-E free diet + 10% safflower oil methyl esters^c^ V-E adequate diet + 10% safflower oil methyl esters^c^ V-E free + 10% oxidized safflower oil methyl esters^c,d^ V-E adequate diet + 10% oxidized safflower oil methyl esters^c,d^	19 days	70% ataxia; 30% mortality 65% ataxia; 20% mortality 90% ataxia; 70% mortality 95% ataxia; 70% mortality
			Exp 3 (20 chicks per group): V-E free diet + 4% safflower oil methyl esters^c^ V-E free diet + 4% safflower oil methyl esters^c^ + 0.3% polar lipid fraction	21 days, from day 8	100% ataxia, 75% mortality 75% ataxia, 60% mortality
			Exp 4 (10 chicks per group): V-E free diet + 4% safflower oil methyl esters^c^ V-E free diet + 4% safflower oil methyl esters^c^ + 0.2% keto-oleate esters^e^	21 days, from day 8	10% ataxia 50% ataxia
			Exp 5 (10 chicks per group): V-E free diet + 4% safflower oil methyl esters^c^ V-E free diet + 4% safflower oil methyl esters^c^ + 0.12% keto-LA esters^f^	21 days, from day 8	50% ataxia 80% ataxia
			Exp 6 (20 chicks per group): V-E free diet + 4% safflower oil methyl esters^c^ V-E free diet + 4% safflower oil methyl esters^c^ + 200 µg/g dicumarol V-E free diet + 4% safflower oil methyl esters^c^ + 400 µg/g dicumarol	21 days, from day 8	60% ataxia, 40% mortality 40% ataxia, 35% mortality 5% ataxia, 5% mortality
Fischer et al.,^22^	Chickens	Unknown	Control diet (“Startena”) V-E basal diet + 150 mg V-E on alternate days V-E free diet + 8% LA	5, 10 or 15 days	Development of autoflourescent granules, Acid phosphatase reaction products and electron-dense bodies in brain capillaries by day 10 of V-E def + 8% LA group
Kokatnur et al.,^27^	Chickens	1 week/male	*Exp 1 (12 chicks per group, except last group, n* *=* *11):* V-E free + 10% corn oil diet V-E free + 10% corn oil + 0.25% 12-oxo-cis-9-octadecenoic acid V-E free + 10% corn oil + 0.25% 12-oxo-octadecanoic acid V-E free + 10% corn oil + 0.25% 12-oxo-trans-10-octadecenoic acid V-E free + 10% corn oil diet + 0.25% 12-oxo-cis-9-octadecenoic acid methyl ester	7 days	Encephalomalacia seen in: 17% 100% 42% 42% 91%
			*Exp 2 (n* *=* *6 per group):* V-E free + 2.5%, 5% or 10% corn oil diet V-E free + 2.5%, 5%, or 10% corn oil + 0.05% 12-oxo-cis-9-octadecenoic acid V-E free + 2.5%, 5%, or 10% corn oil + 0.1% 12-oxo-cis-9-octadecenoic acid V-E free + 2.5%, 5%, or 10% corn oil corn oil + 0.25% 12-oxo-cis-9-octadecenoic acid	7 days	Positive symptoms of Encephalomalacia seen most frequently when corn oil was at 10% and keto-acids at 0.25%

^a^A starter diet lacking vitamin E but containing Fleischmann yeast as source of antioxidants and 5% lard (5% w/w) as the primary source of fat was used for the first 5 days (prior to experimental diet randomization) to achieve normal growth

^b^Oxidation of the oil was achieved by heating it at 145 °C for 24 h

^c^Chicks were started on a 4% oil diet on day 1 after hatching, and switched to the 10% diet from days 8 to 19. Vitamin E content of the V-E + diet was 1 µg/g

^d^Oxidation of the oil methyl ester was achieved by heating it at 145 °C for 3 h

^e^Oleic acid-derived keto-esters containing 96% 8-oxo-octadecenoate, 9-oxo-octadecenoate,10-oxo-octadecenoate, and 11-oxo-octadecenoate

^f^LA-derived keto-esters containing 91% 13-oxo-9,11 and 9-oxo-10,12 – octadecadienoate

Dam et al. reported that 2-day-old chicks fed a V-E deficient diet containing 1.5% ethyl-LA (weight percent) from the 2nd day of life onwards, died from encephalomalacia within 28 days (11 out of 12 chicks), compared to chicks fed a V-E deficient diet containing ethyl-ALA (0 out of 12) or a V-E deficient basal diet lacking both LA and ALA (0 out of 12).^21^ In a follow-up study, the authors found that a V-E deficient diet containing 1.5% ethyl-AA induced encephalomalacia faster than a V-E deficient diet containing 1.5% ethyl-LA or lard (mixed source of LA and AA).^24^ These studies demonstrated that when V-E is absent from the diet, AA and to a lesser extent LA, damage the brain of young chicks. ALA caused no damage.

Follow-up studies in chickens demonstrated that oxidized LA metabolites (i.e., OXLAMs) were the likely cause of encephalomalacia, and that this effect was linked by maternal dietary LA content. Bartov and Bornstein reported that maternal intake of a 4% soybean oil (relatively high in LA) or 4% tallow (low in LA) diet containing 5 or 25 mg/kg α-tocopherol acetate each, from 6 to 11 months of age, reduced egg-yolk LA concentration in the tallow group (independent of V-E dietary content), and decreased V-E concentration in yolk of both groups fed the tallow and soybean diets containing low α-tocopherol acetate (5 mg/kg).^23^ Chicks from each maternal group were then fed a V-E free diet containing 4% or 10% oxidized safflower oil high in LA (~70%) for 31 days to induce encephalomalacia. Oxidation was achieved by heating the oil at 145 °C for 24 h.

The authors reported that regardless of safflower oil content of the chick diet (4 or 10% level), chicks born to hens that were fed the 4% soybean oil diet containing low (5 mg/kg) α-tocopherol acetate levels experienced a significantly higher frequency of ataxia and mortality compared to chicks born hens fed 4% soybean oil containing 25 mg/kg α-tocopherol, or beef tallow containing either 5 or 25 mg/kg α-tocopherol.^23^ Hence, a high LA maternal diet low in V-E accelerated the development of ataxia and encephalomalacia-related mortality in chick offspring maintained on a V-E free diet containing oxidized safflower oil. Lowering maternal dietary LA or supplementing it with V-E was protective.

Budowski et al. performed a series of experiments to understand the cause of ataxia and mortaility linked to encephalomalacia.^25^ They fed a V-E free diet containing oxidized safflower oil or non-heated safflower oil methyl esters at 4% or 10% (w/w) to chicks for 2 to 3 weeks. At 4% oil content, the incidence of ataxia and mortality were higher in the group fed the non-heated safflower oil compared to the group fed oxidized safflower oil. At 10%, ataxia and mortality were higher in the group fed oxidized safflower compared to the group fed non-heated safflower oil. The findings suggest that in the absence of V-E, oxidized safflower oil at 10% (w/w) induced more neurological deficits than at 4%. Also, the fact that ataxia and death were observed in the group fed 4% non-heated safflower oil, suggests the absence of dietary V-E might induce oil auto-oxidation of LA (the main PUFA in safflower oil). It is not known whether the OXLAM species generated by auto-oxidation due to the absence of V-E in the diet, differ from those generated by heating. Notably, this study did not include a control group provided with V-E in the diet.

To test whether V-E was protective, the authors provided 1-day-old male chicks with non-heated or heated 10% (w/w) safflower oil methyl esters lacking or containing 1 µg/kg V-E for 19 days.^25^ Ataxia and mortality were relatively high in the groups fed non-heated oils lacking or containing V-E (65–70% ataxia; 20–30% mortality). In the groups provided with oxidized oil with or without V-E, the incidence of ataxia and mortality were much increased (90–95% ataxia, 70% mortality), irrespective of V-E content. It is possible that higher doses of V-E might have been more effective in reducing mortality and the incidence of ataxia by limiting auto-oxidation, particularly in the non-heated safflower oil group.

The authors also introduced the polar fraction (at 0.3% w/w) to the V-E free diet containing 4% safflower oil, and found that it induced ataxia and increased mortality compared to the same background diet lacking the polar extracts.^25^ Because oleic acid and LA are the main unsaturated fatty acids in safflower oil, the authors introduced oleic acid and LA derived ketone esters (i.e., oxidized fatty acids) at 0.2% and 0.12%, respectively, to the same V-E free diets containing 4% safflower oil. They found that the incidence of ataxia was 50% and 80% with the oleic acid and LA keto-esters, respectively, and thus higher than the diets lacking these oxidized compounds. A separate study by Kokatnur et al. showed that the addition of 0.25% (w/w) 12-oxo-cos-9-octadecenoic acid, 12-oxo-octadecanoic acid, 12-oxo-trans-10-octadecenoic acid, or 12-oxo-cos-9-octadecenoic acid methyl ester to a V-E deficient basal diet containing 10% corn oil, induced encephalomalacia in 42–100% of chickens compared to 17% in those fed the basal diet.^27^ The authors also determined that as little as 0.05% of ketone fatty acids induced encephalomalacia and that this effect was exacerbated by adding more corn oil to the diet (from 2.5 to 10% w/w).^27^ These studies provide direct evidence that OXLAMs, and to a lesser extent oxidized oleic acid metabolites, induce ataxia and mortality due to encephalomalacia in chickens.

In the Budowski et al. study, the administration of the anti-coagulant dicumarol to a V-E free diet containing 4% safflower oil reduced the incidence of ataxia and mortality,^25^ suggesting that the neurotoxic effects of OXLAMs may be caused by blood coagulation and disturbances in the brain’s microvasculature. This is in agreement with a study by Fischer et al. who showed morphologic changes in cerebral vasculature, characteristic of pre-ceroid formation in adult chickens fed an 8% LA diet lacking V-E, compared to chickens on a similar diet containing V-E.^22^ Fully developed ceroids have been linked to neurodegeneration.^28^

## Effects of LA or OXLAMs on rodents

The few studies that explored the direct role of LA and its metabolites in rodents have shown that LA is involved in regulating brain immunity, seizure threshold, and neuronal signaling, and that some of these effects are mediated by its oxidized metabolites (i.e., OXLAMs).

In rats, LA lowering was found to attenuate LPS-induced neuroinflammation, reduce brain concentration of pro-inflammatory lipid autacoids and increase brain levels of anti-inflammatory lipid mediators. Taha et al. reported that compared to a “high” 5.2% energy LA diet, chronic consumption of a “low” 0.4% energy LA diet for 15–18 weeks, reduced the incorporation rate of radiolabeled AA into the brain and prevented the increase in brain AA-derived prostaglandin E_2_ concentration and COX-2 activity induced by 2-day lipopolysaccharide (LPS) administration into the 4th ventricle.^29^ Two independent studies reported that dietary LA lowering (from 5.2% to 0.4% energy) reduced brain concentrations of pro-inflammatory AA-derived eicosanoids (e.g., 5-Hydroxyeicosatetraenoic acid) and increased concentrations of anti-inflammatory EPA or DHA derived metabolites.^30,31^ This may partially explain why LA lowering increased resilience to LPS-induced neuroinflammation in the Taha et al. study.^29^

Acute administration of LA was reported to confer seizure protection in rats. When infused intravenously at a dose of 0.028 mg/kg, it decreased the incidence of tonic-clonic convulsions by 3-fold, but increased the incidence of cortical spike-wave discharges by 1.5 to 2 folds.^32^ Voskuyl et al. showed that intravenous administration of 56–64 mg/kg LA raised cortical focal and generalized seizure thresholds by 3-fold and 1.3–9.0-fold, respectively, in a cortical stimulation seizure model compared to saline and oleic acid controls.^33^ The effect of chronic LA administration on seizure thresholds has not been tested.

Recent studies suggest that the effects of LA in the brain are likely mediated by OXLAMs. Hennebelle et al. showed that OXLAMs are increased in various rat brain regions following acute ischemic injury, possibly due to their involvement in the response to ischemic brain injury.^34^ Direct application of LA itself to rat hippocampal slices did not alter paired-pulse facilitation within soma or dendrites, whereas LA-derived 13-hydroxyoxtadecadieonic acid (13-HODE) increased somatic paired pulse facilitation.^34^ Although the Hennebelle et al. study raises the possibility that LA’s effects on the brain might be mediated by OXLAMs,^34^ it only tested one OXLAM (13-HODE). Future studies should address the isolated and collective effects of other hydrox, epoxy, dihydroxy, trihydroxy, and ketone metabolites of LA on neurotransmission and other brain processes.

**Fig. 1:**
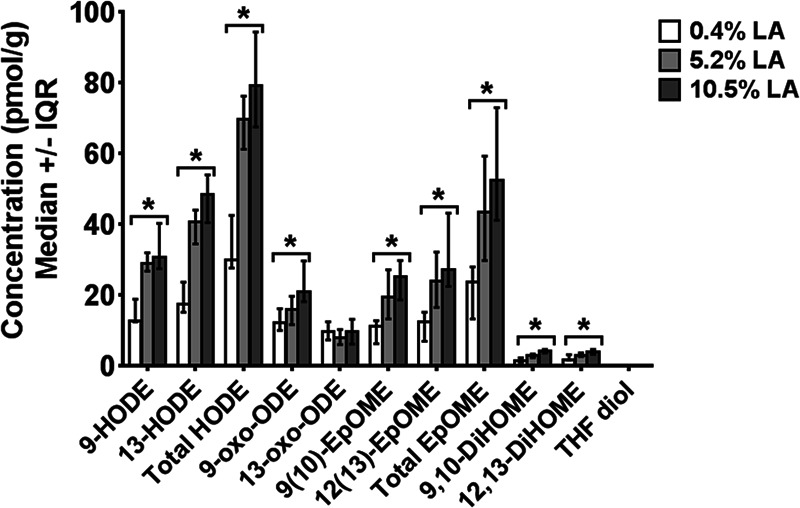
Effect of increasing dietary linoleic acid (LA) on unesterified oxidized linoleic acid metabolite (OXLAM) concentrations in rat cerebral cortex. Adapted from Taha et al.,^31^ *EpOME* epoxyoctadecamonoenoic acid, *THF* tetrahydrofuran-diols, *EKODE* epoxyketooctadecenoic acid, *HODE* hydroxyoctadecadienoic acid, *oxo-ODE* Oxo-octadecadienoic acid, *DiHOME* dihydroxyoctadecamonoenoic acid, *TriHOME* trihydroxyoctadecamonoenoic acid. Data are graphed as median and interquartile interval (IQR) representing the 25th and 75th percentiles. *p < 0.05 compared to 0.4% LA group by Kruskall–Wallis test.

Although OXLAMs are known to be abundant in rodent brain,^31,34^ it is not known whether they are preferentially formed in the brain from LA, or incorporated directly from circulating OXLAMs provided in the diet. This issue was recently addressed by two studies. Taha et al. fed rats a graded LA diet (0.4%, 5.2%, and 10.4% energy) for 15–18 weeks, and showed that increasing dietary LA increased cerebral cortex and cerebellum OXLAM concentrations in a dose-dependent manner (Fig. 1).^31^ In a follow up study, Ramsden et al. fed mice a high LA diet (17% energy), or a low LA diet (4% energy) with or without oxidized corn oil as a source of OXLAMs.^35^ The authors found no evidence of dietary OXLAM incorporation into the brain, but consistent with a previous report, the high LA diet increased brain OXLAM concentrations compared to the low LA diet with or without OXLAMs.^35^ These studies suggest that dietary OXLAMs are not readily incorporated into rodent (mouse) brain, and that LA is the main source of OXLAMs in the brain. Behavioral studies on dietary OXLAMs in rodents are yet to be performed, as has been done previously in chickens. Notably, although the chicken studies outlined in Table 1 showed an effect of OXLAMs on behavior, none assessed their bioavailability. In rodents, dietary LA hydroperoxides were reported to degrade into aldehydes in the stomach, raising the possibility that the reported effects of OXLAMs on the brain might be mediated by their degradation products.^36^

## Effects of LA or OXLAMs on humans

Most of the clinical evidence on the effects of LA on the brain stem from epidemiological studies. One prospective cohort study reported an inverse association between high PUFA intake and mortality linked to neurodegenerative disease.^37^ Although it is difficult to establish causation with such studies, the same study, found no association between LA on neurodegenerative disorders after adjusting for confounders using multi-variate analysis, but reported a significant 4–15% risk reduction in neurodegeneration with increased AA, ALA, or EPA and DHA intake.^37^ Randomized controlled trials are needed to confirm these findings.

Ramsden et al. reported that dietary LA lowering from 7% to 2% energy combined with a high EPA and DHA diet (~1.5 g per day) for 12 weeks, reduced migraine frequency and improved quality of life in patients with drug-resistant migraines.^38,39^ Lowering dietary LA without changing EPA and DHA did not significantly alter migraine frequency or improve quality of life.^38,39^ Although this study did not have a parallel group that received EPA and DHA alone, it provides evidence of a potential synergistic benefit to lowering LA and increasing EPA and DHA simultaneously.^40^ It is possible that longer-term LA lowering regimens might reduce migraine frequency as well, although this remains to be tested.

Several epidemiological studies explored the role of LA on neurodevelopment. Like other dietary fatty acids, LA accumulates in breast milk, which is the primary source of infant nutrition for the first few months months of life.^41^ Thus, consistent with the increase in dietary LA in the food supply, breastmilk LA composition has increased from 7% to 12% of total fatty acids between 1970 and 2000.^41,42^ The 12% composition value corresponds to 8% energy, which exceeds the minimum 1–2% energy required for developing infants by 4–8-fold.^2^

Overall, studies have shown an adverse effect of maternal breast milk or dietary LA on neurodevelopment. One study reported that a high maternal breast milk LA percent composition (>9.7% of fatty acids) was associated with reduced motor and cognitive scores in 2- to 3-year-old infants.^43^ In the same cohort, maternal breastmilk LA percent composition was associated with reduced verbal IQ at 5 to 6 years of age.^44^ In fact, children breastfed with the highest levels of LA had cognitive scores comparable to children who were never breastfed.^43^ These effects were independent of AA or DHA breast milk composition, suggesting a direct impact of excess breast milk LA (and hence maternal intake) on brain development. Consistent with these findings, Lassek and Gaulin found an inverse correlation between breast milk LA percent composition and cognitive scores in 15-year-old children, suggesting a long-lasting impact of maternal LA on offspring cognitive skills.^45^ Steenweg-de Graaff et al. also reported a significant positive association between maternal plasma LA composition measured at mid-pregnancy and the risk of autistic traits in children at the age of 6 years.^46^ A more recent study found that prenatal intake of diets high in the ratio of LA to ALA, was associated with a 2-fold increase in the risk of delayed psychomotor and mental developmental in 6 months infants.^47^

## Conclusion

Pre-clinical and clinical studies dispel previous assumptions that LA is a benign fatty acid in the brain. On the contrary, when present in excess and chronically, it induces ataxia in chickens, promotes neuroinflammation in rats and is linked to abnormal neurodevelopment in humans. LA administered acutely to rats confers seizure protection, although its chronic effects on seizures has not been tested.

Recent animal studies suggest that the effects of LA might be mediated by OXLAMs generated from LA entering the brain. Although OXLAMs are present in the diet,^48^ the extent of their bioavailability and accumulation in the brain from dietary sources appears to be minimal.^35,36^ However, further assessment of their degradation products on brain neurophysiology and other organs is warranted.

Chronic consumption of low LA diets might protect the brain against inflammation, as evidenced by rat studies showing an anti-inflammatory lipidome in the brains of rats fed a low LA diet,^29–31^ and human studies showing that LA lowering combined with EPA and DHA reduced headache frequency in patients with drug-resistant migraines.^38,39^

In conclusion, this review presents evidence that excess LA in the food supply might adversely affect the brain. The potential benefit of LA lowering merits detailed evaluation in well-designed and adequately-powered clinical studies, to test whether this translates into tangible reductions in the risk of neurodegenerative disorders and neurodevelopmental abnormalities at a population level.

## Supplementary information

figure permission
