# Publisher Correction: Linoleic acid–good or bad for the brain?

**DOI:** 10.1038/s41538-020-0066-4

**Published:** 2020-04-01

**Authors:** Ameer Y. Taha

**Affiliations:** Department of Food Science and Technology, College of Agriculture and Environmental Sciences, University of California, Davis, CA 95616 USA

**Keywords:** Neurochemistry, Oils

Correction to: *npj Science of Food* 10.1038/s41538-019-0061-9, published online 2 January 2020

In the original version of this Article, “atypical” in the Abstract was mistakenly changed to “a typical” at proof checking. This has now been corrected in the HTML and PDF versions of the Article.

